# Accounting for the mortality benefit of drug-eluting stents in percutaneous coronary intervention: a comparison of methods in a retrospective cohort study

**DOI:** 10.1186/1741-7015-9-78

**Published:** 2011-06-24

**Authors:** Robert W Yeh, Malini Chandra, Charles E McCulloch, Alan S Go

**Affiliations:** 1 Cardiology Division, GRB800, Department of Medicine, Massachusetts General Hospital, Harvard Medical School, Boston, MA 02114, USA; 2 Division of Research, Kaiser Permanente of Northern California, 2000 Broadway Street, 3rdFloor, Oakland, CA 94612, USA; 3 Division of Biostatistics, Department of Epidemiology and Biostatistics, University of California, San Francisco, 185 Berry Street, Suite 5700, San Francisco, CA 94107, USA

## Abstract

**Background:**

Drug-eluting stents (DES) reduce rates of restenosis compared with bare metal stents (BMS). A number of observational studies have also found lower rates of mortality and non-fatal myocardial infarction with DES compared with BMS, findings not observed in randomized clinical trials. In order to explore reasons for this discrepancy, we compared outcomes after percutaneous coronary intervention (PCI) with DES or BMS by multiple statistical methods.

**Methods:**

We compared short-term rates of all-cause mortality and myocardial infarction for patients undergoing PCI with DES or BMS using propensity-score adjustment, propensity-score matching, and a stent-era comparison in a large, integrated health system between 1998 and 2007. For the propensity-score adjustment and stent era comparisons, we used multivariable logistic regression to assess the association of stent type with outcomes. We used McNemar's Chi-square test to compare outcomes for propensity-score matching.

**Results:**

Between 1998 and 2007, 35,438 PCIs with stenting were performed among health plan members (53.9% DES and 46.1% BMS). After propensity-score adjustment, DES was associated with significantly lower rates of death at 30 days (OR 0.49, 95% CI 0.39 - 0.63, *P *< 0.001) and one year (OR 0.58, 95% CI 0.49 - 0.68, *P *< 0.001), and a lower rate of myocardial infarction at one year (OR 0.72, 95% CI 0.59 - 0.87, *P *< 0.001). Thirty day and one year mortality were also lower with DES after propensity-score matching. However, a stent era comparison, which eliminates potential confounding by indication, showed no difference in death or myocardial infarction for DES and BMS, similar to results from randomized trials.

**Conclusions:**

Although propensity-score methods suggested a mortality benefit with DES, consistent with prior observational studies, a stent era comparison failed to support this conclusion. Unobserved factors influencing stent selection in observational studies likely account for the observed mortality benefit of DES not seen in randomized clinical trials.

## Background

The comparison of alternative treatments has long been a primary mission of both randomized trials and observational studies. With the commitment of $1.1 billion in support of comparative effectiveness research in the American Recovery and Reinvestment Act of 2009, the number of studies comparing different drugs, devices, techniques and systems will undoubtedly increase dramatically [1]. While randomized clinical trials are likely to remain the gold standard for comparing alternative treatments, observational studies should continue to have significant, if not leading, roles in comparative effectiveness research moving forward, particularly in light of recommendations to prioritize assessments of community-based interventions within populations traditionally underrepresented in clinical trials [2].

However, observational studies are subject to a number of limitations, foremost among them the potential for unmeasured variables that confound results. While a number of methods directed at assessing causal effects and eliminating confounding have been developed, few clinical studies describe reasons for the specific choice of method used, and fewer present multiple methods to help corroborate findings[3,4].

Observational studies comparing drug-eluting stents (DES) and bare metal stents (BMS) for percutaneous coronary intervention (PCI) have consistently shown lower mortality and myocardial infarction associated with DES [5-20], findings not seen in randomized clinical trials [21-27]. We applied three common methods to compare DES to BMS within a large observational study population to: 1) determine whether mortality benefit for DES was observed in our study population and 2) to identify potential challenges to the application of these methods to compare treatments in the presence of strong treatment selection.

## Methods

We conducted a retrospective dynamic cohort study within Kaiser Permanente of Northern California (KPNC), a large integrated healthcare delivery system caring for > 3.2 million individuals that are broadly representative of the local surrounding and statewide population[28]. All health plan members aged 30 years and older between January 1998 and the end of December 2007 were considered eligible. The study was reviewed by the institutional review board of the Kaiser Division of Research, and requirement for informed consent was waived due to the nature of the study. From this cohort, we identified all PCI procedures using either DES or BMS based on relevant *International Classification of Diseases, Ninth Revision, Clinical Modification *(*ICD-9-CM*) and Current Procedural Terminology (CPT) codes that occurred within health plan and non-health plan hospitals [29]. Consecutive procedures occurring within 7 days of one another were considered part of the same clinical episode. Procedures in which both DES and BMS were used were excluded from the analysis.

### Patient characteristics and coexisting illnesses

Patient demographic information was obtained from health plan electronic databases. Relevant ICD-9 or CPT codes found in hospital discharge databases during the eight years before the procedure date were used to identify prior cardiovascular disease including prior myocardial infarction, prior angina, prior PCI or coronary artery bypass graft (CABG) surgery, history of ischemic stroke and peripheral arterial disease. Prior chronic heart failure was determined based on a validated algorithm using information from hospitalization, outpatient and emergency department diagnostic codes[30].

Relevant data sources were searched for cardiovascular risk factors. Diabetes mellitus was identified from a validated longitudinal Diabetes Registry relying on inpatient and outpatient diabetes diagnoses, receipt of anti-diabetic therapies, and abnormal glycosylated hemoglobin or blood glucose levels [31]. Hypertension was based on serial ambulatory diagnoses or the combination of diagnoses and receipt of anti-hypertensive medications. Chronic lung disease, systemic malignancy, and history of gastrointestinal bleeding were determined based on validated methods [29-35]. The presence and severity of chronic kidney disease was ascertained using the abbreviated four-variable Modification of Diet in Renal Disease (MDRD) equation for estimated glomerular filtration rate based on most recent outpatient determination of serum creatinine within the previous 24 months before the year examined[33].

### Medication usage

Prior observational studies comparing outcomes for DES and BMS have not included the chronic outpatient use of cardioprotective medications, an important potential confounder [6,19,36]. Outpatient medication use within 30 days before PCI was ascertained from health plan pharmacy records for therapies known to lower cardiovascular risk, including beta-blockers [37], angiotensin-converting enzyme (ACE) inhibitors or angiotensin receptor blockers (ARBs),[38] statins [39], and thienopyridines [40]. Outpatient warfarin use was also assessed. More than 90% of patients had a drug benefit that provided strong financial incentives to obtain medications from health plan pharmacies.

### Primary outcomes

Death from any cause occurring within thirty days and one year after the procedure was identified from health plan administrative databases, proxy information, Social Security Administration vital status files, and California state death certificate information[41,42]. Subsequent hospitalized myocardial infarction based on *ICD-9-CM *codes (primary discharge diagnosis coded as 410.x1) within one year was also identified.

### Statistical analysis

Three separate methods were used to compare outcomes after DES and BMS: 1) statistical adjustment based on propensity score decile, 2) propensity-score matching, and 3) a stent era comparison, in which outcomes before and after the introduction of DES in 2003 were compared.

### Method 1: propensity-score adjustment

Because patients were not eligible to receive DES until after April of 2003, only PCI procedures after this date were included in the propensity score methods. A non-parsimonious logistic regression model was used to generate propensity scores for the likelihood of receiving DES based on 23 demographic and clinical variables. Propensity score deciles were created, and logistic regression was performed modeling the association of DES with outcomes, adjusted for propensity score decile (categorical variable) as well as the complete set of patient covariates. To accommodate data from individuals who may have undergone multiple procedures during the study period, robust standard errors accounting for clustering by patient were used.

### Method 2: propensity-score matching

In the second method, one-to-one nearest neighbor matching was performed to compare outcomes after DES and BMS in patients undergoing PCI after April 2003. Caliper width was set at 0.0001 using the STATA program *psmatch2 *(Leuven and Sianesi, 2003) [43]. Adequacy of the match was assessed by estimating the standardized differences between DES and BMS patients for all variables. Outcomes were compared between groups using the McNemar's Chi-square test for matched comparisons.

### Method 3: stent era comparison

**Figure 1:**
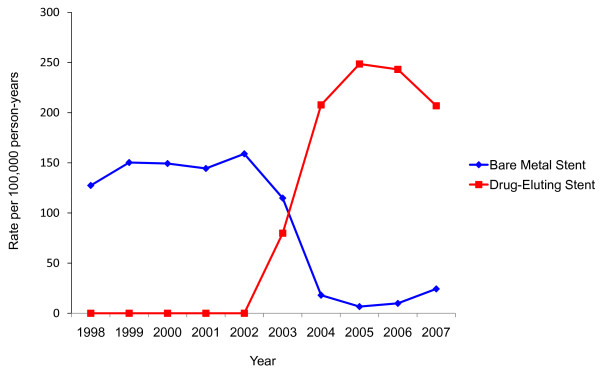
**Rates of percutaneous coronary intervention with drug-eluting and bare metal stents between 1998-2007, per 100,000 person-years**. All stents prior to April 2003 were bare metal, and 88.3% of stents after April 2003 were drug-eluting.

Because DES was not available before 2003 and from 2004 onward DES was used in the vast majority of cases, a comparison of outcomes of PCI prior to and after DES introduction serves as a useful surrogate for assessing the impact of DES (Figure 1) [4,36,44]. Procedures between January 1998 and March 2003 were assigned to the 'BMS era', while those between April 2003 and December 2007 were assigned to the 'DES era'. This variable would be expected to be strongly associated with treatment assignment, and less prone to unmeasured confounding by stent indication. Logistic regression was then performed to determine the association of stent era with outcomes, adjusted for all assessed covariates.

Analyses were performed using STATA (Stata Corp. 2008. Stata Statistical Software: Release 10, College Station, TX: StataCorp LP). A two-sided *P *value of < 0.05 was considered significant for all analyses.

## Results

A total of 35,438 PCIs with stent placement between January 1998 and December 2007 were included. Of these, 19,103 (53.9%) utilized DES and 16,335 (46.1%) utilized BMS. Patients receiving DES were significantly different than those receiving BMS in nearly all baseline characteristics, including having significantly higher rates of hypertensions, diabetes, and congestive heart failure (Table 1). DES patients also had lower rates of presentation with ST-elevation myocardial infarction within 30 days prior to PCI, and were more likely to be receiving outpatient cardioprotective medications including statins, beta-blockers and thienopyridines. Crude-mortality was significantly higher at thirty days (2.2% versus 1.6%, *P *< .001) and one year (5.1% versus 4.6%, *P *= 0.04) after PCI with BMS compared to DES. The rate of myocardial infarction at one year was 4.7% after BMS and 4.3% after DES (*P *= 0.06).

**Table 1:** Characteristics of Patients Undergoing PCI by Stent Type, 1998-2007

	Drug-Eluting Stent	Bare Metal Stent	*P *Value
N	19,103	16,335	
Age (years)	64.1	63.2	< 0.001
Male Sex (%)	70.8	70.6	0.72
Race/Ethnicity (%)			< 0.001
Asian	9.8	7.0	
Black	5.3	5.3	
Hispanic	9.4	8.4	
White, non-Hispanic	65.8	72.1	
Other	9.7	7.3	
Comorbidities (%)			
Diabetes	33.8	26.2	< 0.001
Hypertension	68.5	49.6	< 0.001
Prior MI	13.0	11.1	< 0.001
PCI Indication:			< 0.001
STEMI	16.0	27.5	
NSTEMI	28.0	19.3	
Stable or unstable angina	57.3	54.8	
Prior PCI	19.1	14.1	< 0.001
Prior CABG	5.7	5.3	0.12
Congestive Heart Failure	4.6	3.4	< 0.001
Prior Stroke	1.6	1.2	0.005
Peripheral Arterial Disease	4.3	3.3	< 0.001
Glomerular Filtration Rate			< 0.001
> 90	15.0	16.2	
60 - 89	39.7	33.4	
30 - 59	21.5	15.2	
15 - 30	1.9	1.3	
< 15 or HD	0.7	0.5	
Unknown	21.1	33.3	
Systemic Malignancy	5.7	5.8	0.75
Prior Gastrointestinal Bleeding	2.3	1.8	< 0.001
Chronic Lung Disease	18.3	21.9	< 0.001
Medications			
β-Blockers	50.0	40.1	< 0.001
Clopidogrel	12.7	6.3	< 0.001
Statin	51.7	34.8	< 0.001
ACE-I/ARB	41.9	27.8	< 0.001
Warfarin	3.8	3.0	< 0.001

Abbreviations: MI, myocardial infarction; STEMI, ST-elevation myocardial infarction; NSTEMI, non-ST-elevation myocardial infarction; PCI, percutaneous coronary intervention; CABG, coronary artery bypass graft surgery.

**Figure 2:**
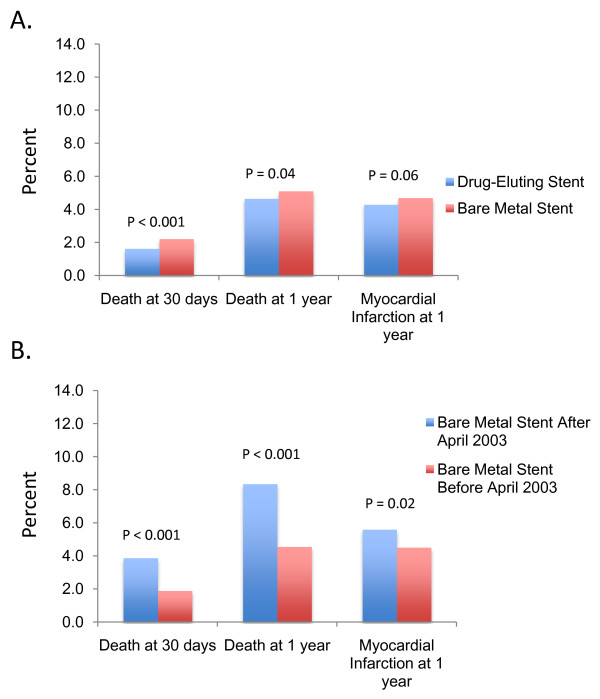
**Unadjusted outcomes after percutaneous coronary intervention**. Outcomes after percutaneous coronary intervention (PCI) with drug-eluting versus bare metal stents for the overall cohort are shown in A. Outcomes after PCI with bare metal stents before and after April 2003 (B). Patients receiving bare metal stents after April 2003 have significantly worse unadjusted outcomes compared to those receiving bare metal stents before April 2003.

Patients receiving BMS after April 2003 were markedly different than those receiving BMS before April 2003, having higher rates of hypertension (65.0% versus 46.8%, *P *< 0.001), congestive heart failure (4.3% versus 3.2%, *P *= 0.008), prior PCI (9.3% versus 5.6%, *P *< 0.001), systemic malignancy (9.3% versus 5.2%, *P *< 0.001) and prior gastrointestinal bleeding requiring hospitalization (3.0% versus 1.7%, *P *< 0.001). Crude-mortality at thirty days and one year and myocardial infarction at one year after PCI were significantly higher in patients receiving BMS after April 2003 compared those receiving BMS before 2003 (Figure 2).

### Association between stent type and outcomes by analytical method

**Figure 3:**
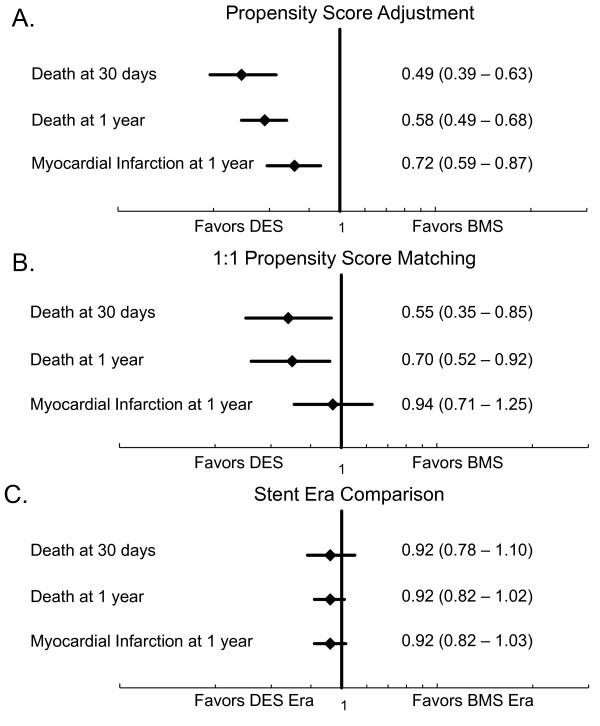
**Association between stent type and 30-day and 1-year mortality, and myocardial infarction at 1 year using different analystical methods**. Results are for (A) propensity-score adjustment; (B) 1:1 propensity score matching; and (C) a stent era comparison. The stent era comparison gives results most consistent with findings from randomized clinical trials.

After propensity-score adjustment, DES was associated with significantly lower mortality at thirty days (OR 0.49, 95% CI 0.39 - 0.63, *P *< 0.001) and one year (OR 0.58, 95% CI 0.49 - 0.68, *P *< 0.001), as well as a lower rate of myocardial infarction at one year (OR 0.72, 95% CI 0.59 - 0.87, *P *< 0.001). After 1:1 propensity-score matching (n = 4,126), DES was again associated with significantly lower mortality at thirty days (OR 0.55, 95% CI 0.35 - 0.85, *P *= 0.005), and at one year (OR 0.70, 95% CI 0.52 - 0.92, *P *= 0.01). Myocardial infarction at one year was similar (OR 0.94, 95% CI 0.71 - 1.25, *P *= 0.67). All standardized differences between groups were < 10%, suggesting an adequate match (Table 2). In the stent era comparison, no differences in mortality were seen when comparing the DES era versus BMS era at thirty days (OR 0.92, 95% CI 0.78 - 1.10, *P *= 0.39) or one year (0.92, 95% CI 0.82 - 1.02, *P *= 0.12), or in the rate of myocardial infarction at one year (OR 0.92, 0.82 - 1.03, *P *= 0.16) (Figure 3).

**Table 2:** Standardized Differences Between BMS and DES Patients Before and After Matching, April 1, 2003 - December 31, 2007

	Standardized Difference Prior to Match	Standardized Difference Post Match
N	(2,518 BMS, 19,101 DES)	(2,063 BMS, 2,063 DES)
Age (years)	-8.3	1.9
Male Sex (%)	-1.7	-6.2
Race/Ethnicity	-11.3	-5.8
Comorbidities (%)		
Diabetes	9.1	3.5
Hypertension	7.5	-3.2
Prior MI	2.7	4.7
PCI Indication:	-21.6	0.2
Prior PCI	6.1	6.1
Prior CABG	2.5	2.5
Congestive Heart Failure	3.8	3.8
Prior Stroke	0.0	0.0
Peripheral Arterial Disease	1.9	1.9
Glomerular Filtration Rate	0.3	0.3
Systemic Malignancy	-13.4	0.4
Prior Gastrointestinal Bleeding	-4.2	-0.6
Chronic Lung Disease	-0.3	7.8
Medications		
β-Blockers	11.9	-1.1
Clopidogrel	8.9	3.4
Statin	12.3	5.3
ACE-I/ARB	9.2	4.1
Warfarin	1.0	3.1

Abbreviations: MI, myocardial infarction; PCI, percutaneous coronary intervention; CABG, coronary artery bypass graft surgery.

## Discussion

We have shown that after the introduction of drug-eluting stents, bare metal stents were reserved for patients with significant comorbidities and high mortality rates. Commonly used propensity-score methods continued to suggest a mortality benefit to DES, while a stent era comparison showed no significant differences in all outcomes for DES and BMS, similar to results from randomized clinical trials.

A number of prior observational studies comparing DES to BMS have suggested reductions in mortality and myocardial infarction associated with DES, the majority of which have utilized propensity-score methods to mitigate potential confounding [5-20]. However, data from randomized trials have not corroborated these findings [21-27]. In a meta-analysis of 22 randomized trials and 34 observational studies, Kirtane et al found no differences in mortality or myocardial infarction in randomized trials, but consistent reductions for both outcomes in observational studies, and attributed these differences, at least in part, to residual confounding by unmeasured differences between patients receiving BMS and DES [23]. Our results are consistent with this conclusion. In our cohort, patients who received BMS after April 2003 had significantly greater comorbidities and unadjusted death and myocardial infarction rates compared to patients who received BMS before this date, suggesting that physicians selectively reserved BMS for a sicker population of patients after DES was introduced. While propensity-score adjustment and matching both attempt to account for such differences, they only account for variables that are assessed. In the presence of such a high degree of patient selection, any residual confounding in this case is likely to lead to the appearance of improved outcomes with DES, as was seen in this study. While propensity scores have been put forward as a method which overcomes some of the limitations of traditional logistic regression, they do not address a primary threat to the validity of such studies - unmeasured confounding.

A comparison of the BMS era (100% BMS) to the DES era (88.3% DES) as a surrogate for a BMS-DES comparison eliminates the unwanted influence of patient selection [45]. Consistent with this, the stent era comparison showed no differences in mortality or myocardial infarction between BMS and DES, most closely approximating results seen in randomized trials. However, because such an analysis compares patients undergoing PCI at two different times, important secular trends may have influenced the results. For example, comparisons of stent eras in the Medicare population and in the New York PCI Registry both suggested lower rates of myocardial infarction in the DES era compared to BMS era [36,44]. However, neither study adjusted for differences in the use of outpatient medications such as statins and beta-blockers, which have increasingly been used in patients with coronary disease over time [46]. In this study, we found significantly higher rates of use of beta blockers, statins, angiotensin-converting enzyme inhibitors, and thienopyridines in the DES era. After accounting for these and other secular trends, no differences in myocardial infarction were observed between DES and BMS patients.

A number of circumstances specific to stent use allowed for the critical examination of these analytical methods. First, the rapid uptake of DES over BMS created a natural experiment allowing use of a stent era comparison. Second, because a large number of patients from randomized clinical trials comparing DES with BMS have been performed, these studies help provide an estimate of the 'truth.' However, in other examples in which the questions of interest may be less well studied, there could be much greater difficulty interpreting disparate results.

## Conclusions

In summary, we have compared outcomes after PCI with either DES or BMS, using three commonly used methods of adjustment in observational studies. Large baseline differences in BMS and DES groups suggested strong treatment selection, which were incompletely adjusted for by propensity-score methods. In the presence of such treatment selection, alternative methods of analysis of observational data should be considered.

## List of abbreviations

ACE: angiotensin-converting enzyme; ARBs: angiotensin receptor blockers; BMS: bare metal stents; CABG: coronary artery bypass graft; CPT: Current Procedural Terminology; DES: drug-eluting stents; ICD-9-CM: International Classification of Diseases: Ninth Edition: Clinical Modification; KPNC: Kaiser Permanente of Northern California; MDRD: modification of diet in renal disease; PCI: percutaneous coronary intervention.

## Competing interests

The authors declare that they have no competing interests.

## Authors' contributions

RWY conceived of the original idea for the study, conducted statistical analyses, and drafted the manuscript. MC assisted with analysis of the data and critically reviewed the manuscript. CEC advised on biostatistical methodology of the study and provided critical revisions of the manuscript. ASG supervised in the conception and design of the study and provided critical revisions of the manuscript. All authors read and approved the final manuscript.

## Acknowledgements and funding

No additional acknowledgements.

This work was funded by institutional funds from the Massachusetts General Hospital and Kaiser Permanente of Northern California.

## Pre-publication history

The pre-publication history for this paper can be accessed here:

http://www.biomedcentral.com/1741-7015/9/78/prepub
